# Synthesis of 2a,8b-Dihydrocyclobuta[*a*]naphthalene-3,4-diones

**DOI:** 10.3762/bjoc.6.76

**Published:** 2010-07-13

**Authors:** Kerstin Schmidt, Paul Margaretha

**Affiliations:** 1 Chemistry Department, University of Hamburg, Martin-Luther-King Platz 6, D-20146 Hamburg, Germany

**Keywords:** cyclobutenes, photocycloaddition, quinone monoacetals

## Abstract

On irradiation (λ = 350 nm) in neat hex-1-yne, naphthalene-1,2-dione monoacetals **1** afford mixtures of pentacyclic photodimers and up to 25% (isolated yield) of mixed photocycloadducts **2**. Careful acidic hydrolysis of the acetal function of **2** gives the title compounds **3**, the overall sequence representing a first approach to a (formal) [2 + 2] photocycloadduct of a 1,2-naphthoquinone to an alkyne.

## Introduction

The behaviour of excited 1,2- and 1,4-quinones towards ground-state molecules differs greatly. Whereas the former typically react via H-abstraction by an excited carbonyl group [1], the latter smoothly undergo [2 + 2] cycloaddition to alkenes to afford cyclobutane-type products [2]. Very recently we reported the use of 1,2-dihydro-1,1-dimethoxynaphthalen-2-ones **1** as protected precursors for the synthesis of both photocyclodimers and ketene-photocycloadducts of 1,2-naphtoquinones [3–4]. Here we report the preparation of – novel – 2a,8b-dihydrocyclobuta[*a*]naphthalene-3,4-diones, i.e. (formal) 1,2-naphthoquinone + alkyne [2 + 2] cycloadducts.

## Results

**Scheme 1:**
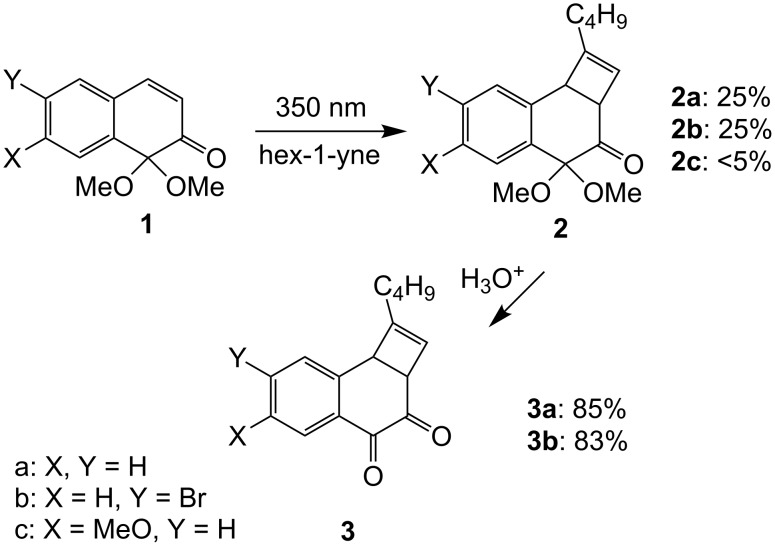
Synthesis of 2a,8b-dihydrocyclobuta[*a*]naphthalene-3,4-diones.

Irradiation of **1** in the presence of alkynes affords the – known [3] – pentacyclic dimers and variable amounts (0–33%) of enone + alkyne cycloadducts as indicated by ^1^H NMR spectroscopy. The yields of mixed cycloadducts with many alkynes (3,3-dimethylbut-1-yne, trimethylsilylacetylene, 3-[(trimethylsilyl)oxy]prop-1-yne or hex-3-yne were invariably low (<5%). Moderately higher yields (15–25%) were obtained using hex-1-yne in either benzene or acetonitrile as solvent. Best results were obtained using hex-1-yne, both as reaction partner and as solvent. Thus, irradiation of either **1a** or **1b** in neat hex-1-yne affords a mixture of the corresponding dimeric dibenzophenylenediones (two regioisomers [3], 67–70%) and up to 30–33% of cycloadducts **2a** or **2b**, respectively. Compounds **2** can easily be isolated by chromatography (25% isolated yield) as they exhibit much higher *R*_f_-values than the corresponding dimers. In contrast, naphthalenone **1c** under the same conditions only affords <5% of **2c**. Hydrolysis of cycloadducts **2** in a two phase mixture (CH_2_Cl_2_, aq HCl) at r.t. [5] leads to quantitative deprotection of the acetal function as indicated by ^1^H NMR spectroscopy to afford compounds **3a** or **3b**, respectively (Scheme 1). Compounds **3** are also easy to purify by chromatography (83–85% isolated yield) which is greatly assisted by the fact that they are easily detectable on account of their yellow colour.

## Discussion

At first glance, the (relatively) low yield of mixed cycloadduct formation from excited **1** and alkynes seems disappointing. Nevertheless, one should bear in mind that *a*) dimer formation on irradiation of phenyl-conjugated enones, e.g., 3-phenylcyclohex-2-enone, is not suppressed even in neat alkenes as solvent [6], as these compounds tend to associate via π–π-stacking, and *b*) radical additions to alkynes usually proceed with significantly lower relative rates (30–50%) than those to the corresponding alkenes [7]. Taking these findings and the observed regioselectivity of the cycloaddition into consideration, the maximum relative yield (33%) of compounds **2a** or **2b** at total conversion of starting material is acceptable. Moreover, the fact that hydrolysis of the cycloadducts proceeds quantitatively, then the overall yields in the preparation of the – novel – 1,2-naphthoquinone + alkyne cycloadducts even becomes satisfactory. In the same experiment with **1c**, the MeO-group apparently tends to increase the efficiency in photodimerization vs mixed photocycloaddition, otherwise there is no obvious explanation for this result.

## Experimental

**1. General.** Acetals **1** were synthesized according to [8]. Both **1b**, m.p. 60–62 °C, and **1c**, m.p. 76–78 °C, originally described as oils, solidified on standing. Hex-1-yne was commercially available. Photolyses were conducted in a *Rayonet RPR-100* photoreactor equipped with (16) 350 nm lamps. Column chromatography (CC) was carried out with silica gel 60 (Merck; 230–400 mesh). ^1^H and ^13^C NMR spectra (including 2D plots) were recorded with a *Bruker WM-500* instrument at 500.13 and 125.8 MHz, resp., in CDCl_3_, δ in ppm, *J* in Hz.

**2. Photolyses.** Ar-Degassed solns. of **1** (1 mmol) in hex-1-yne (10 ml) were irradiated for 15 h up to total conversion (monitoring by TLC). After evaporation of the excess alkyne, the crude mixtures were analyzed by ^1^H NMR in order to determine the crude yield. CC (SiO_2_, pentane/Et_2_O 6:1) gave the photocycloadducts **2**. *1-Butyl-3,4-dihydro-4,4-dimethoxy-2a*H*,8b*H-*cyclobuta[a]naphthalen-3-one* (**2a**): 72 mg (25%), colourless oil, *R*_f_ = 0.65. ^1^H NMR: 7.70 (d, *J* = 8.4, 1H); 7.36 (t, *J* = 8.4, 1H); 7.30 (m, 2H); 5.97 (s, 1H); 4.52 (d, *J* = 4.6, 1H); 4.00 (bs, 1H); 3.53 & 3.00 (s, 3H); 2.16 (t, *J* = 7.0, 2H); 1.52 (m, 2H); 1.38 (m, 2H); 0.92 (t, *J* = 6.9, 3H). ^13^C NMR: 203.1 (s); 156.2 (s); 137.3 (s); 134.5 (s); 129.1 (d); 128.6 (d); 128.4 (d); 127.8 (d); 125.2 (d); 99.1 (s); 51.0 (q); 50.1 (d); 49.2 (q); 48.3 (d); 30.2 (t); 28.6 (t); 22.5 (t); 14.2 (q). Anal. Calcd for C_18_H_22_O_3_: C, 75.50; H, 7.74. Found: C, 75.43; H, 7.78. *7-Bromo-1-butyl-3,4-dihydro-4,4-dimethoxy-2a*H*,8b*H-*cyclobuta[a]naphthalen-3-one* (**2b**): 91 mg (25%), light yellow solid, m.p. 50 – 52 °C, *R*_f_ = 0.61. ^1^H NMR: 7.55 (d, *J* = 8.4, 1H); 7.42 (s, 1H); 7.41 (d, *J* = 8.4, 1H); 5.96 (s, 1H); 4.46 (d, *J* = 4.6, 1H); 3.95 (bs, 1H); 3.52 & 2.97 (s, 3H); 2.16 (t, *J* = 7.0, 2H); 1.52 (m, 2H); 1.38 (m, 2H); 0.93 (t, *J* = 6.9, 3H). ^13^C NMR: 203.2 (s); 156.2 (s); 135.9 (s); 135.5 (s); 133.0 (s); 131.9 (d); 129.9 (d); 129.8 (d); 125.8 (d); 99.1 (s); 51.1 (q); 50.2 (d); 49.1 (q); 48.2 (d); 30.2 (t); 28.6 (t); 22.5 (t); 14.2 (q). Anal. Calcd for C_18_H_21_BrO_3_: C, 59.19; H 5.79. Found: C, 59.22; H, 5.82.

**3. Hydrolyses.** To a soln. of the acetal **2** (0.2 mmol) in CH_2_Cl_2_ (2 ml), was added 8N HCl (1.5 ml) and the mixture stirred for 5 h at room temperature. The org. phase was washed with sat. aq NaCl, dried (MgSO_4_) and the residue (100% conversion to product from ^1^H NMR) purified by CC (SiO_2_, pentane/Et_2_O 1:1) to afford the diketones **3**. *1-Butyl-2a,8b-dihydrocyclobuta[a]naphthalene-3,4-dione* (**3a**): 37 mg (85%), viscous yellow oil, *R*_f_ = 0.45. ^1^H NMR: 8.06 (d, *J* = 8.5, 1H); 7.62 (t, *J* = 8.5, 1H); 7.42 (t, *J* = 8.5, 1H); 7.37 (d, *J* = 8.5, 1H); 5.72 (s, 1H); 4.25 (d, *J* = 3.2, 1H); 4.16 (bs, 1H); 1.97 (m, 2H); 1.40 (m, 2H); 1.26 (m, 2H); 0.83 (t, *J* = 6.9, 3H). ^13^C NMR: 196.2 (s); 184.5 (s); 164.1 (s); 144.2 (s); 137.1 (s); 134.5 (d); 130.1 (d); 128.4 (d); 127.8 (d); 122.5 (d); 48.5 (d); 46.4 (d); 28.8 (t); 28.0 (t); 27.4 (t); 22.4 (q). Anal. Calcd for C_16_H_16_O_2_: C, 79.97; H, 6.71. Found: C, 79.92; H, 6.85. *7-Bromo-1-butyl-2a,8b-dihydrocyclobuta[a]naphthalene-3,4-dione* (**3b**): 48 mg (83%), viscous yellow oil, *R*_f_ = 0.41. ^1^H NMR: 7.94 (d, *J* = 8.5, 1H); 7.58 (d, *J* = 8.5, 1H); 7.53 (s, 1H); 5.74 (s, H); 4.19 (d, *J* = 3.1, 1H); 4.15 (bs, 1H); 1.99 (m, 2H); 1.40 (m, 2H); 1.26 (m, 2H); 0.84 (t, *J* = 6.9, 3H). ^13^C NMR: 196.1 (s); 184.6 (s); 164.2 (s); 144.2 (s); 137.1 (s); 134.5 (d); 130.1 (s); 128.4 (d); 127.8 (d); 122.5 (d); 48.6 (d); 46.3 (d); 28.8 (t); 28.0 (t); 27.4 (t); 22.4 (q). Anal. Calcd for C_16_H_15_BrO_2_: C, 60.21; H, 4.71. Found: C, 60.13; H, 4.77.
